# Association between obstructive sleep apnea and quality of life in Korean middle-aged people: a cross-sectional study

**DOI:** 10.1186/s12955-025-02390-y

**Published:** 2025-06-15

**Authors:** Jiwon Kim, Min Jeong Joo, Jae Yong Shin, Chung-Mo Nam, Eun-Cheol Park

**Affiliations:** 1 Yonsei University Graduate School of Public Health, Seoul, Republic of Korea; 2 Department of Public Health, Graduate School, Yonsei University, Seoul, Republic of Korea; 3 Institute of Health Services Research, Yonsei University, Seoul, Republic of Korea; 4 Department of Preventive Medicine & Institute of Health Services Research, Yonsei University College of Medicine, 50 Yonsei-to, Seodaemun-gu, Seoul, 03722 Republic of Korea

**Keywords:** Middle-aged and older adults in South Korea, Obstructive sleep apnea, Quality of life

## Abstract

**Background:**

Obstructive sleep apnea (OSA), a serious sleep disorder, can lead to comorbidities and decreased quality of life if untreated. Poor sleep quality from OSA affects concentration, cognitive function, and mental health, contributing to conditions such as depression and anxiety. OSA prevalence increases with age, and middle-aged adults are particularly at risk owing to age-related social and physical changes. Enhancing sleep quality is essential for improving overall quality of life. The aim of this study was to investigate the relationship between OSA risk and quality of life among middle-aged and older adults in South Korea.

**Methods:**

This study utilized data from the 8th Korea National Health and Nutrition Examination Survey for 2019 and 2021 to investigate the relationship between OSA and health-related quality of life (HRQoL). After excluding missing values, the analysis included 8,109 adults aged ≥ 40 years. The primary variable of interest was OSA risk, with HRQoL measured using the HINT-8 index. Chi-square tests, binary logistic regression, and multinomial logistic regression analyses were conducted to examine the association between OSA and HRQoL.

**Results:**

In this study of 4,831 participants, 59.6% were classified as high-risk for OSA. Low quality of life was significantly associated with high-risk OSA (adjusted odds ratio [aOR], 1.17: 95% confidence interval [CI], 1.03–1.33). Subgroup analyses showed higher odds of low quality of life in high-risk OSA individuals, particularly among those not engaged in economic activity (aOR, 1.39; 95% CI, 1.15–1.67), those who consumed alcohol (aOR, 1.24; 95% CI, 1.03–1.49), and those with limited physical activity (aOR, 1.21; 95% CI, 1.03–1.43). Higher OSA risk correlated with poorer quality of life, especially in the lowest quality of life category (aOR, 2.49; 95% CI 1.18–3.43).

**Conclusions:**

The study found that middle-aged and older adults in South Korea at high risk for OSA had a lower quality of life than those at low risk. High-risk individuals who were economically inactive, consumed alcohol, or engaged in low physical activity also showed a lower quality of life. Future research should focus on accurately measuring OSA and further exploring its impact on quality of life in this population.

## Background

Obstructive sleep apnea (OSA) is a sleep disorder characterized by repeated interruptions in breathing during sleep, causing temporary decreases in blood oxygen levels and increases in carbon dioxide level [1]. Often mistaken for “simple” snoring, OSA is marked by frequent breathing disturbances that disrupt the sleep cycle and reduce deep sleep time. If left untreated, it can result in a chronic state of sleep deprivation [2], comorbidities [3], and increased accidents [4], affecting social functioning [5], mental health [6], and physical activities [7].

The characteristics of OSA that can affect sleep quality can also affect an individual’s quality of life and satisfaction [8–13] Factors related to OSA are essential elements that determine quality of life [14]. The World Health Organization defines quality of life as how individuals perceive and respond to their health status and other non-medical aspects of their lives [15]. This can be represented as the sum of various objectively measurable conditions that an individual experiences [16]. Although improving subjective life satisfaction is often examined in other disciplines [17], in general, a higher quality of life measured using objective indicators tends to correspond to higher subjective life satisfaction [18, 19].

Middle-aged adults can experience physical aging, menopause, multiple illnesses, and changes in economic capacity, vitality, cognitive function, and social roles [20]. Among these changes, factors associated with OSA risk, such as obesity [21] and hypertension [22], frequently occur in middle-aged adults. These health issues not only increase the risk of developing OSA but also complicate its management, highlighting the need for regular monitoring and timely interventions. Moreover, the impact of OSA in middle-aged adults can extend beyond physical health, affecting mental well-being and quality of life, making early detection and management crucial in this demographic.

The prevalence of OSA varies between 9% and 38%, with higher rates observed among adult men [23]. Although studies have examined the relationship between OSA and subjective sleep quality [24] and depression [25, 26], research on the relationship between OSA and quality of life remains limited. For example, one study on obese children has shown that health-related quality of life(HRQoL) is more strongly associated with self-reported sleep experiences than with the presence of OSA [27]. As the South Korean population is increasingly characterized as an “aging” society, new evidence is needed to help maintain and improve the quality of life of older adults. Therefore, the aim of this study was to explore the relationship between OSA risk and HRQoL among middle-aged and older adults in South Korea.

## Methods

Data were obtained from the Korea National Health and Nutrition Examination Survey (KNHANES) conducted in 2019 and 2021. The KNHANES is a nationwide statutory survey conducted annually by the Korea Disease Control and Prevention Agency to assess the health behaviors, chronic disease prevalence, and dietary and nutritional intake of the Korean population. The sampling framework used the most recent data from the Population and Housing Census to ensure the representative sampling of individuals aged 1 year and older residing in South Korea. The results of the KNHANES are utilized to develop and improve health policies and used in various research activities aimed at promoting health and preventing diseases. Additionally, the data help compare health levels between countries and fulfill requirements of the World Health Organization and Organization for Economic Cooperation and Development. This study did not require ethical approval as the KNHANES adheres to the Declaration of Helsinki.

### Study population

**Fig. 1:**
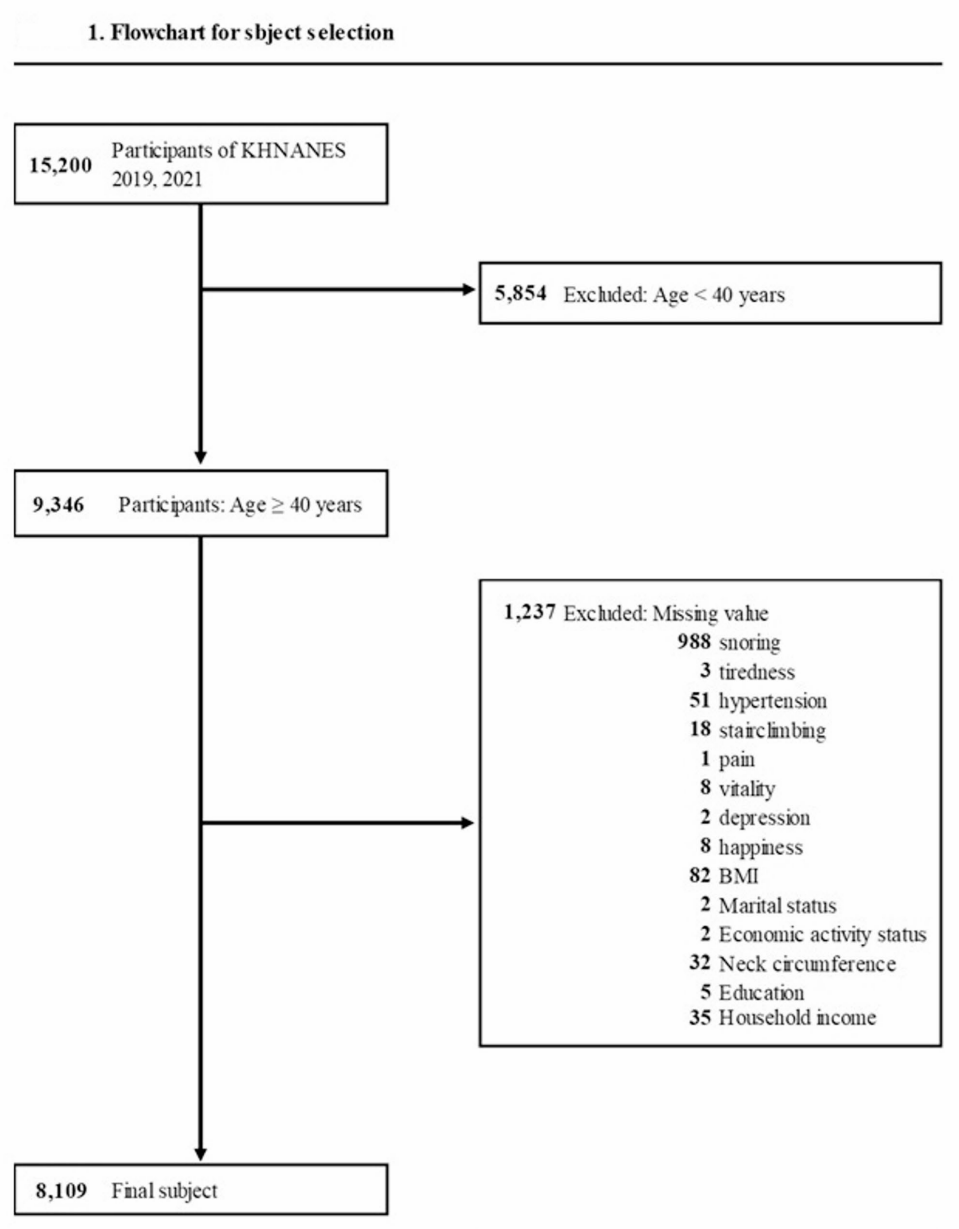
Flowchart for subject selection

This study used data from the KNHANES conducted in 2019 and 2021, which included 15,200 participants. As the focus was on middle-aged and older adults, individuals under 40 years old (*n* = 5,854) were excluded. Additionally, participants lacking information on factors relevant to identifying OSA symptoms (*n* = 1,161) and those with missing data (*n* = 76) were excluded. Finally, 8,109 participants (3,504 male and 4,605 female) were included in the study (Fig. 1).

### Variables

The presence of OSA risk was evaluated using the STOP-Bang score [28]. In 2019, KNHANES introduced STOP-Bang as a new screening tool for OSA. The STOP-Bang questionnaire is a simple, useful, and user-friendly tool for OSA screening. It is widely used as a highly sensitive screening tool for OSA [29]. The acronym STOP-Bang represents the initials of each symptom or physical characteristic related to OSA: Snoring, Tiredness, Observed apnea, and high Blood pressure(STOP) - BMI, Age, Neck circumference, and Gender(Bang). Each question could be answered with “yes” or “no,” where “yes” responses were assigned a value of 1 and “no” responses a value of 0, with a total score range of 0 to 8.

Sleep-related symptoms were assessed using the following questions: (1) Do you snore loudly enough to be heard through closed doors or walls? (2) Do you often feel tired or sleepy during the day? (3) Has anyone observed you stop breathing during your sleep?

Blood pressure was measured using an internationally validated non-mercury automatic blood pressure monitor (Microlife WatchBP Office AFIB). BMI was calculated as weight (kg) divided by height (m²). Although the original STOP-Bang questionnaire assigns a point for a BMI of ≥ 35, this study adjusted the threshold to ≥ 30 based on the BMI criteria for Asians [30, 31]. Neck circumference was measured below the thyroid cartilage using a Lufkin W606PM device. The STOP-Bang questionnaire assigns 1 point for a neck circumference of ≥ 41 cm in women and ≥ 43 cm in men. However, this threshold has been found to be too high to effectively screen Korean patients with OSA. Therefore, we applied the Korean-specific criteria suggested in a previous study that evaluated the sensitivity and specificity of these cut-off values in Korean patients. Accordingly, for men, a neck circumference of less than 36.3 cm was classified as normal, and 36.3 cm or more as thick. For women, a neck circumference of less than 32.3 cm was considered normal, and 32.3 cm or more as thick [32]. The risk of OSA was determined based on global cutoff values [33], with a score of ≤ 2 indicating a low risk of OSA and ≥ 3 indicating a high risk of OSA.

The dependent variable of our study, quality of life, was assessed using the Health-related Quality of Life Instrument with eight items (HINT-8), as utilized in the KNHANES [34]. The HINT-8 consists of eight items (climbing stairs, pain, vitality, work, depression, memory, sleep, and happiness) and four levels (no problem, mild, moderate, and severe problem), capable of representing 65,536 health states. The HINT-8 score ranges from 0.132 (44444444, the worst possible state) to 1.000 (11111111, the best possible state), with the index derived from a previously developed value set. In this study, participants were divided into two groups based on the median HINT-8 score of the sample (0.813): those with scores above the median were classified as having a high quality of life and those with scores at or below the median were classified as having a low quality of life.

Independent variables included region (metropolitan, urban, rural), marital status (single, separated/divorced/widowed, married), household income (quartiles), education level (elementary school or less, middle school graduate, high school graduate, college graduate or higher), and employment status (employed, unemployed). Health behavior-related factors included alcohol consumption (less than 1 time per month, 1–4 times per month, more than 2 times per week), smoking status (non-smoker, ex-smoker, smoker), physical activity by Metabolic Equivalent of Tasks index [35] (low, mid, high), and number of chronic diseases diagnosed (none, 1–2, ≥ 3).

### Statistical analysis

A descriptive analysis was performed using the chi-square test to examine the distribution of the general characteristics of the study population. After considering potentially confounding variables, multiple logistic regression analyses were performed to investigate the association between OSA and quality of life among middle-aged and older adults. Subgroup analyses were performed to investigate the combined effects of each covariate on quality of life and OSA. Odds ratios (ORs) and 95% confidence intervals (CIs) were calculated to compare the data of participants with depressive symptoms. The variables were clustered, stratified, and weighted to enhance the representativeness and accuracy of the target population, ensuring that the findings reflect the characteristics of the South Korean population [36]. All analyses were performed using SAS (version 9.4; SAS Institute, Cary, NC, USA). Differences were considered statistically significant at *p* < 0.05.

## Results

Table 1 presents the general characteristics of the study population. In total, 3,504 participants were included in the study. Among these participants, 4,831 (59.6%) were classified as high-risk for OSA. Of the 4,079 individuals classified as having a low quality of life based on the HINT-8 median score, 2,570 (63%) were in the high-risk OSA group.

**Table 1:** General characteristics of the study population

Variables		Quality of life (HINT-8)								
		Total			High			Low		
		*N*	%	*N*		%	*N*		%	*P*-value
Total (*N* = 8, 109)		8,109	100	4,030		50	4,079		50	
Obstructive Sleep Apnea risk										< 0.0001
	Low	**3,278**	40.4	1,769		54.0	1,509		46.0	
	High	**4,831**	59.6	2,261		46.8	2,570		53.2	
Marital status										< 0.0001
	Married	**6,229**	76.8	3339		53.6	2890		46.4	
	Separated/divorced/bereaved	**1,535**	18.9	520		33.9	1015		66.1	
	Single	**345**	4.3	171		49.6	174		50.4	
Region										0.0004
	Metropolitan	**3,331**	41.1	1671		50.2	1660		49.8	
	Urban	**2,846**	35.1	1471		51.7	1375		48.3	
	Rural	**1,932**	23.8	888		46.0	1044		54.0	
Education										< 0.0001
	Under elementary school	**1,992**	24.6	630		31.6	1,362		68.4	
	Middle school	**1,020**	12.6	437		42.8	583		57.2	
	High school	**2,605**	32.1	1,420		54.5	1,185		45.5	
	University and over	**2,492**	30.7	1,543		61.9	949		38.1	
Economic activity status										< 0.0001
	No	**3,307**	40.8	1350		40.8	1957		59.2	
	Yes	**4,802**	59.2	2680		55.8	2122		44.2	
Smoking										< 0.0001
	Non-smoker	**4,828**	59.5	2230		46.2	2598		53.8	
	Ex-smoker	**2,061**	25.4	1,171		56.8	890		43.2	
	Smoker	**1,220**	15.0	629		51.6	591		48.4	
Alcohol intake										< 0.0001
	Less than 1 time per month	**2,916**	36.0	1229		42.1	1687		57.9	
	1–4 times per month	**3,587**	44.2	1928		53.7	1659		46.3	
	more than 2 times per week	**1,606**	19.8	873		54.4	733		45.6	
Household income(quartile)										< 0.0001
	Low	**1,985**	24.5	814		41.0	1171		59.0	
	Mid-low	**2,005**	24.7	975		48.6	1030		51.4	
	Mid-high	**2,071**	25.5	1102		53.2	969		46.8	
	High	**2,048**	25.3	1139		55.6	909		44.4	
Physical exercise(METs^a^)										< 0.0001
	High	**712**	8.8	444		62.4	268		37.6	
	Mid	**3,109**	38.3	1674		53.8	1435		46.2	
	Low	**4,288**	52.9	1912		44.6	2376		55.4	
Chronic disease^b^										< 0.0001
	None	**4,020**	49.6	2288		56.9	1732		43.1	
	1–2	**3,716**	45.8	1626		43.8	2090		56.2	
	3≤	**373**	4.6	116		31.1	257		68.9	

a: High: > 3,000 MET-min (≥ 3 days of vigorous activity ≥ 1 day; 1,500 min walking ≥ 7 days; or ≥ 7 days of moderate and vigorous activity); Mid: ≥ 600 MET-min (≥ 3 days of vigorous activity ≥ 1 day; 30 min of moderate activity ≥ 5 days, or ≥ 5 days of walking); Low: No moderate or vigorous activity, or below the criteria for moderate and vigorous groups

b: Number of physician-diagnosed conditions: Dyslipidemia, Stroke, Myocardial infarction, Angina, Pulmonary tuberculosis, Asthma, Sinusitis, Allergic rhinitis, Diabetes

Table 2 shows the results related to factors associated with quality of life and high OSA risk after adjusting for all covariates. There was a significant association between a high risk of OSA and low quality of life (adjusted odds ratio [aOR], 1.17; 95% confidence interval [CI], 1.03–1.33).

**Table 2:** Results of factors associated with HINT-8 and high OSA risk

Variables^a^		Quality of life (HINT-8: Low)			
		aOR	95% CI		
Obstructive Sleep Apnea risk					
	Low	1.00			
	High	1.17	(1.03	-	1.33)

a: Adjusted for Marital status, Region, Education, Economic activity status, Smoking, Alcohol intake, Household income, Physical exercise(METs), Chronic disease

Table 3 presents the results of subgroup analyses stratified by each independent variable after adjusting for all other covariates. Among those not engaged in economic activity, the likelihood of having a lower quality of life was higher in the high-risk OSA group than in the low-risk OSA group (aOR, 1.39; 95% CI, 1.15–1.67). In the group that consumed alcohol 1–4 times per month, the high-risk OSA group had a higher likelihood of low quality of life than the low-risk OSA group (aOR, 1.24; 95% CI, 1.03–1.49). Similarly, in the group with low physical activity, the high-risk OSA group had a higher likelihood of low quality of life than the low-risk OSA group (aOR, 1.21; 95% CI, 1.03–1.43).

**Table 3:** The results of subgroup analysis stratified by independent variables

Variables		Quality of life (HINT-8:Low)				
		OSA risk				
		Low	High			
		OR	aOR	95% CI		
**Marital status**						
	Married	1.00	1.15	(0.99	-	1.33)
	Separated/divorced/bereaved	1.00	1.41	(1.07	-	1.86)
	Single	1.00	1.10	(0.66	-	1.85)
**Region**						
	Metropolitan	1.00	1.07	(0.89	-	1.29)
	Urban	1.00	1.25	(1.01	-	1.54)
	Rural	1.00	1.26	(0.97	-	1.63)
**Education**						
	Under elementary school	1.00	1.29	(1.00	-	1.68)
	Middle school	1.00	1.32	(0.96	-	1.82)
	High school	1.00	1.20	(0.98	-	1.47)
	University and over	1.00	1.04	(0.83	-	1.30)
**Economic activity status**						
	No	1.00	1.39	(1.15	-	1.67)
	Yes	1.00	1.06	(0.91	-	1.24)
**Smoking**						
	Non-smoker	1.00	1.22	(1.05	-	1.42)
	Ex-smoker	1.00	1.11	(0.83	-	1.48)
	Smoker	1.00	1.07	(0.77	-	1.50)
**Alcohol intake**						
	Less than 1 time per month	1.00	1.23	(1.00	-	1.50)
	1–4 times per month	1.00	1.24	(1.03	-	1.49)
	more than 2 times per week	1.00	1.01	(0.74	-	1.39)
**Household income(quartile)**						
	Low	1.00	1.58	(1.23	-	2.04)
	Mid-low	1.00	1.18	(0.92	-	1.52)
	Mid-high	1.00	1.08	(0.85	-	1.37)
	High	1.00	0.99	(0.77	-	1.29)
**Physical exercise(METs**^**a**^)						
	High	1.00	0.96	(0.63	-	1.45)
	Mid	1.00	1.17	(0.95	-	1.43)
	Low	1.00	1.21	(1.03	-	1.43)
**Chronic disease** ^**b**^						
	None	1.00	1.06	(0.90	-	1.25)
	1–2	1.00	1.34	(1.12	-	1.61)
	3≤	1.00	1.07	(0.50	-	2.28)

a: High: > 3,000 MET-min (≥ 3 days of vigorous activity ≥ 1 day; 1,500 min walking ≥ 7 days; or ≥ 7 days of moderate and vigorous activity); Mid: ≥ 600 MET-min (≥ 3 days of vigorous activity ≥ 1 day; 30 min of moderate activity ≥ 5 days, or ≥ 5 days of walking); Low: No moderate or vigorous activity, or below the criteria for moderate and vigorous groups

b: Number of physician-diagnosed conditions: Dyslipidemia, Stroke, Myocardial infarction, Angina, Pulmonary tuberculosis, Asthma, Sinusitis, Allergic rhinitis, Diabetes

Table 4 shows the association between low quality of life and the severity of OSA. The higher the risk of OSA, the higher the likelihood of lower quality of life. However, in the intermediate group, this association was non-significant (aOR, 1.43; 95% CI, 1.22–1.68).

**Table 4:** Association between quality of life(HINT-8) and each severity level of obstructive sleep apnea

Variables		Quality of life (HINT-8:Low)				
		aOR	95% CI			
Obstructive Sleep Apnea risk						
	**Low**	1.00				
	**Intermediate**	1.04	(0.91	-	1.19)	
	**High**	1.43	(1.22	-	1.68)	

When quality of life was categorized into five groups (good, middle-high, middle, middle-low, low), individuals in the high-risk OSA group were more likely to have poorer quality of life than those in the low-risk OSA group. This association was particularly significant for the lowest quality of life group, where the odds ratio was notably higher (Low: aOR, 2.49; 95% CI, 1.18–3.43) (Table 5).

**Table 5:**
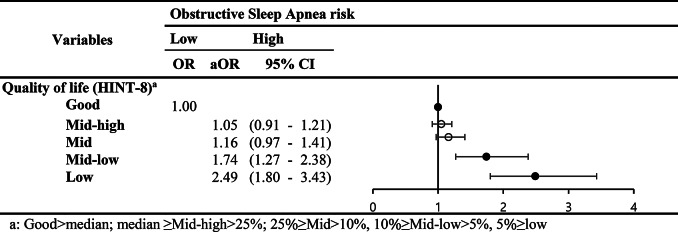
The results of subgroup analysis stratified by quality of life (HINT-8)

## Discussion

This cross-sectional study utilized 2 years of data from the KNHANES to investigate the association between the risk of OSA and quality of life among middle-aged and older adults in South Korea. After adjusting for potential covariates, the quality of life in the high-risk OSA group was significantly lower than that in the low-risk OSA group. Specifically, the risk of lower quality of life was higher in those who were not economically active, consumed alcohol, and had low physical activity levels when comparing the high-risk OSA group to the low-risk OSA group.

Those at a high risk for OSA experience excessive sleepiness and sleep deprivation, which negatively affect job performance [5], leading to decreased productivity [37, 38] and an increased risk of unwanted job loss [39]. The high-risk OSA group may have lower abilities to obtain and maintain employment than the low-risk OSA group [40]. Furthermore, individuals who are not engaged in professional activities are more likely to be exposed to OSA-related risk factors, such as alcohol consumption, obesity, and low physical activity [41]. In addition, economically inactive individuals may be situated in environments where the negative impact of OSA on quality of life is more pronounced. Limited access to healthcare, lower health literacy, and psychological stress associated with unemployment or financial insecurity may hinder the recognition and management of OSA symptoms [42]. These conditions can ultimately lead to poorer quality of life. This supports our finding that among those who are not economically active, the high-risk OSA group may have a lower quality of life than the low-risk OSA group. However, even those who are not engaged in economic activities can maintain a good quality of life if they lead a healthy lifestyle [43, 44]. This suggests that while economic inactivity may increase vulnerability to poor quality of life in individuals at high risk for OSA, lifestyle and psychosocial factors such as dietary patterns and social support may serve as protective buffers and should be considered in future research and intervention strategies.

In the group of individuals who consumed alcohol, those at a high risk for OSA had a poorer quality of life than those at a low risk for OSA. Excessive alcohol consumption can increase the risk of developing OSA [45], with the frequency and amount of alcohol intake being related to OSA risk [46]. Several studies have examined the mechanisms underlying the relationship between alcohol consumption and OSA, such as the impact of alcohol on the respiratory system [47] and its potential to induce apnea [48]. Thus, alcohol consumption can be a risk factor for OSA, and abstaining from alcohol may be an important intervention for individuals with OSA to improve their condition. Continuous alcohol consumption in the high-risk OSA group perpetuated an unhealthy cycle, aligning with our findings that OSA was associated with a lower quality of life. Moreover, alcohol is associated with mental health problems such as depression and fatigue, both of which can further deteriorate perceived quality of life [49]. In this context, alcohol use may act as both a behavioral risk factor for OSA and an independent determinant of poorer quality of life.

Low levels of physical activity are highly associated with OSA risk, partly due to their relationship with metabolic disorders, obesity, and cardiovascular diseases [50, 51]. Additionally, physical inactivity has been linked to increased fatigue, depressed mood, and poor sleep quality, which may exacerbate OSA symptoms and reduce overall quality of life. Notably, physical activity has been found to improve mood and quality of life in individuals with OSA, regardless of symptom severity [52]. Therefore, our finding that individuals at high risk for OSA with low physical activity had a lower quality of life than those at low risk is consistent with the existing literature and highlights the importance of promoting physical activity as a potential intervention for improving outcomes in this population.

The results of this study indicate a significant association between the risk of OSA and lower quality of life among middle-aged and older adults. However, it is important to acknowledge several limitations of the study. First, its cross-sectional design means it can only identify associations between variables and cannot establish causality. Additional longitudinal or experimental studies are needed to confirm causal relationships. Second, the risk of OSA and quality of life were measured using the STOP-Bang index and HINT-8, respectively. These self-reported surveys might be subject to response bias and inaccuracies owing to the memory limitations of the participants and their potential unwillingness to report accurately. Additionally, the STOP-Bang index indicates the risk of OSA rather than its prevalence. Third, the STOP-Bang index is not an objective diagnostic method for OSA but is primarily used as a screening tool instead of performing polysomnography. Therefore, further studies using polysomnography data are necessary. Fourth, although covariates that might influence the dependent variable were controlled for, unmeasured confounding variables may have affected the results. Specifically, the absence of variables such as dietary patterns and social support, which are known to be associated with both OSA risk and quality of life, may have introduced residual confounding. Finally, as we applied BMI and neck circumference thresholds based on Asian standards, different results may be obtained using the original STOP-Bang criteria.

Despite these limitations, our study has several strengths. First, it used a representative sample, reflecting the general situation in South Korea, and can, thus, be used to inform better health policies. Second, the study used reliable measures, such as the STOP-BANG score and HINT-8, to assess OSA risk and quality of life. These reliable results can help shape more effective public health policies and interventions to address quality of life issues among middle-aged and older adults.

## Conclusions

The results of this study indicate that middle-aged and older adults in South Korea who are at high risk for OSA have a lower quality of life than those at low risk. Additionally, those at high risk for OSA who are economically inactive, consume alcohol, or engage in low levels of physical activity may experience even lower quality of life. Future research is needed to accurately measure OSA and further elucidate its association with quality of life among middle-aged and older adults, and should also consider examining individual dimensions of quality of life to better understand the specific ways in which OSA affects daily functioning.

## Data Availability

No datasets were generated or analysed during the current study.

## Abbreviations

OSA: Obstructive sleep apnea
KNHANES: Korea National Health and Nutrition Examination Survey
HRQoL: Health-related quality of life
aOR: adjusted odds ratio
CI: Confidence interval
BMI: Body mass index
STOP-Bang: Snoring, Tiredness, Observed apnea, and high Blood pressure
BMI: Age, Neck circumference, and Gender
HINT-8: Health-related Quality of Life Instrument with eight items
